# Evaluation of suicidal risk and severity in psychiatric emergency departments of different hospitals of the Autonomous Community of Madrid

**DOI:** 10.1192/j.eurpsy.2025.1457

**Published:** 2025-08-26

**Authors:** A. Martínez Pillado, L. M. Navío García, C. Blanco Londoño, M. Don Pedro Montes, C. M. Rodríguez González, R. Alvarez Garcia

**Affiliations:** 1 PSYCHIATRY DEPARTMENT, HOSPITAL REY JUAN CARLOS; 2 PSYCHIATRY DEPARTMENT, HOSPITAL FUNDACION JIMENEZ DIAZ; 3 MEDICINE, UNIVERSIDAD REY JUAN CARLOS, MADRID, Spain

## Abstract

**Introduction:**

Suicide is a public health problem whose impact is increasing every year, causing more than 700,000 deaths per year globally (WHO). This includes Spain, where there is an upward trend with differences according to sociodemographic areas. The use of an appropriate suicide risk assessment scale could be a fundamental tool in psychiatric emergencies.

**Objectives:**

The main objective: to analyze the differences in suicidal risk in different geographical areas of the CAM, using the C-SSRS in psychiatric emergencies for which patients from a capital area of Madrid (HUFJD) were compared with a peripheral area (HURJC).

**Methods:**

Observational, analytical, cohort and multicenter study on a total of 4375 patients attended between July 2023 and January 2024 in the psychiatric emergency department of a hospital in the center of Madrid (Fundación Jiménez Díaz) and in a peripheral hospital (Hospital Rey Juan Carlos). Since the first date, these services have implemented the Columbia Scale to Assess Suicide Risk (C-SSRS) as an assessment tool in their clinical records.

**Results:**

Of the patients, 34.95% were at high risk of suicide (moderate and high risk levels according to the C-SSRS), 8.2% were at low risk and 56.9% were not at suicidal risk. Statistically significant differences were found in high suicidal risk, being higher in the peripheral hospital (Hospital Rey Juan Carlos 40.2% vs Fundación Jiménez Díaz 30.8%; p<0.000). In addition, logistic regression shows a ratio: 1.32 times higher high suicidal risk in women, 3.26 times in patients who self-harm, 1.5 times in those without socio-familial support and up to 1.99 times in those under 65 years of age.

**Conclusions:**

This study confirms the importance of sociodemographic data in the incidence of increased suicide risk, among which the geographic area of residence stands out, with a difference of more than 10% in the risk of high suicide risk between the periphery (40.2% in HURJC) and the capital (30.8% in HUFJD) of the CAM, which could be related to the different socioeconomic levels in both areas. Other sociodemographic factors that increase suicidal risk are: age between 18 and 65 years (with higher incidence between 18 and 25 years), female sex, and lack of sociofamily support. In addition to these factors, self-injury and previous suicide attempts are important suicide risk factors.The availability of a tool such as the C-SSRS that can be administered to all patients attending psychiatric emergency departments seems a fundamental strategy to assess suicidal risk and severity. Its implementation in all hospitals would provide more objective data to develop lines of research, which is essential given the great impact that suicide has at a global level and its worrying trends that are continually on the rise.

**Disclosure of Interest:**

None Declared

